# High concentration of MSG alters antioxidant defence system in lobster cockroach *Nauphoeta cinerea* (Blattodea: Blaberidae)

**DOI:** 10.1186/s13104-020-05056-8

**Published:** 2020-04-16

**Authors:** Blessing A. Afolabi, Olawande C. Olagoke

**Affiliations:** 1 Departamento de Bioquímica, Instituto de Ciências Básicas da Saúde, Universidade Federal do Rio Grande do Sul, Rua Ramiro Barcelos 2600-Anexo, 90035-003 Porto Alegre, RS Brazil; 2 Departamento de Bioquímica e Biologia Molecular, Centro de Ciências Naturais e Exatas (CCNE), Universidade Federal de Santa Maria, 97105-900 Santa Maria, RS Brazil; 3 Department of Biochemistry, Bowen University, Iwo, Osun State Nigeria

**Keywords:** *Nauphoeta cinerea*, Monosodium glutamate, Catalase, Glutathione-S-Transferase, Total thiol, Acetylcholinesterase, Oxidative stress, Food additive

## Abstract

**Objective:**

Monosodium glutamate (MSG) is a food additive that has been shown to be toxic to rodents at high concentrations. The available studies in *Drosophila melanogaster* suggest that MSG toxicity depends on concentration and gender, thus the safety of MSG as a food enhancer still requires further investigation. We have documented impaired locomotor activity and altered oxidative stress markers in cockroaches co-exposed to methylmercury and monosodium glutamate (MSG). We herein examined the susceptibility of *Nauphoeta cinerea* to high and low concentrations (4% and 1%) of MSG, while monitoring the activities of acetylcholinesterase (AChE), as well as markers of oxidative stress and antioxidant activity over 30 days.

**Results:**

There was no significant alteration in the parameters assessed at 1% MSG while 4% MSG caused an increase in the activity of reactive oxygen and nitrogen species, with a corresponding reduction in the activities of acetylcholinesterase, glutathione-S-transferase and catalase, suggesting the capacity of MSG to alter redox homeostasis in *Nauphoeta cinerea*.

## Introduction

Monosodium glutamate [(MSG), (e-number E621)], is known to be one of the most widely used food enhancer [1], apart from the common table salt. MSG is a derivative of glutamate and an abundant non-essential amino acid in nature. The average daily intake of MSG in humans is estimated to be 300–1000 mg, although this varies in different countries [2]. Organizations and nutritionists endorse it and also reiterate its safety in humans [3], however, debates persist over the health implications of MSG consumption. Several animal studies have linked MSG with oxidative stress and toxicity to the liver, kidney or reproductive organs [4–7]. Recently, studies have shown an adaptive response to oxidative stress in *Drosophila melanogaster* and a reduction in life span after short exposure to MSG diet [8]. However, another study in *D. melanogaster* showed that MSG could be safe at extremely low concentrations, though high concentration caused alterations in catalase activity [9].

The cockroach is a promising model being utilized for toxicological and behavioural experiments [10–13]. The biophysical principles of nervous system function in insects and mammals are analogous because they possess similar neurotransmitters, albeit their distributions vary widely [14, 15]. Thus, as an alternative to conventional animal models, the present study investigated the effect of MSG compounded diet on acetylcholinesterase, as well as markers of oxidative stress and antioxidant activity in *N. cinerea*.

## Main text

### Materials and methods

#### Chemicals

Monosodium glutamate (MSG 99% purity Ajinomoto^®^) was gotten from Carrefour supermarket in Santa Maria-RS, Brazil. Sigma Aldrich (St. Louis, MO, USA) supplied sodium chloride, glutathione, 1-chloro-2,4 dinitrobenzene (CDNB), 5ʹ,5ʹ-dithiobis-2-nitrobenzoic acid (DTNB), acetylthiocholine iodide, and hydrogen peroxide (H_2_O_2_). All other chemicals were of high purity while the water was glass distilled.

#### Cockroach husbandry and experimental protocol

*Nauphoeta cinerea* was obtained from Departamento de Bioquímica e Biologia Molecular, CCNE, Universidade Federal de Santa Maria, Brasil. Plastic boxes (45.5 cm × 40.2 cm x 29.5 cm) were used to rear the nymphs before some were randomly selected into transparent boxes (25 cm x 17 cm x 7.5 cm) for the experiment. They were acclimatized in the transparent box for 10 days before exposure to MSG diet and maintained at a temperature and humidity of ± 26 °C and ± 75% respectively. Nymphs of *N. cinerea* were selected for this study because it is the most active developmental stage and their age can be estimated from the length. In addition, the adults rarely increase in length. The nymphs used for both studies had lengths ranging from 1.09 to 1.76 cm at the start and 1.12 to 1.86 cm at the end of the experiment.

The insects could freely access water and standard dog food as composed in Afolabi et al. [10] during rearing and adaptation. For the diet, MSG (1% or 4%) or NaCl (1% or 4%) was mixed with a 20 g diet containing 10 g milled corn flour, 7 g wheat flour, 2 g granulated sugar, 0.5 g casein, 0.4 g powder milk and 0.1 g table salt and stored at -20 °C. Both studies consisted of 3 groups of 30 nymphs each, nymphs from the first study were exposed to basal diet (control), 4% NaCl and 4% MSG, while nymphs for the second study were exposed to basal diet (control), 1% NaCl and 1% MSG for 30 days respectively. These periods of exposure and concentration were chosen based on prior food preference studies (1, 2, 3 and 4% of NaCl and MSG) which showed nymphs of cockroaches consumed more diet compounded with 4% MSG, hence a lower concentration was also chosen. The nymphs consumed about 42 mg/day of the MSG diet (Data not shown).

#### Determination of acetylcholinesterase (AchE), oxidative stress and antioxidant activity markers

Following the period of exposure, nymphs were anaesthetized on ice, the heads carefully excised, weighed and homogenized in ice-cold 0.1 M phosphate buffer, pH 7.4, using 100 mg head: 1 mL buffer and centrifuged at 2500× *g* for 10 min at 4 °C to obtain a supernatant that was utilized for biochemical estimations. The protein content was estimated using UV–visible 1650 PC Spectrophotometer (Shimadzu) at 280 nm.

Acetylcholinesterase activity was estimated with the method of Ellman et al. [16]. We used 110 μL distilled water, 50 μL 0.1 M potassium phosphate buffer (pH 7.4), 30 μL sample (0.8 mg/ml protein), 20 μL 10 mM DTNB, and 20 μL 8 mM acetylthiocholine. The Spectra Max plate reader was set at 412 nm, 24 min (30 s interval) and results were expressed as μmolthiocholine formed/min/mg protein.

The quantification of 2′,7′-dichlorofluorescein (DCFH) oxidation to assess the intracellular level of RONS, a general index of oxidative stress was done [17]. The mixture consisted of 150 μL 0.1 M potassium phosphate buffer (pH7.4), 40μL distilled water, 5μL 200 μM DCFH-DA and 5 μL tissue sample (0.2 mg/ml protein). The emission of DCF fluorescence resulting from DCFH oxidation was analyzed for 45 min (30 s intervals) at 488 and 525 nm, excitation and emission wavelengths respectively, using a spectra Max plate reader (Molecular Devices, CA, USA). The rate of DCF formation was expressed as arbitrary units.

Thiobarbituric acid reactive substances (TBARS) were measured according to the established method [18]. Heads of cockroaches were homogenized in the ratio 1 mg to 5 μL 0.1 M potassium phosphate buffer (pH 7.4). 200 μL of the resultant supernatant and 400 μL of stock reagent (equal ratio of trichloroacetic acid (10%, w/v) and 2-thiobarbituric acid (0.75%, w/v) in 0.1 M Hcl) were incubated (95 °C, 60 min), cooled, centrifuged (8000 x g, 10 min) and read at 532 nm. Weight of cockroach head was used to normalize results that were expressed as nmol MDA/g tissue.

Catalase activity measures the rate of disappearance of hydrogen peroxide. The reaction medium consisted of 850 μL 0.05 M potassium phosphate buffer (pH 7.0), 60 μL sample (0.8 mg/ml protein), and 90 μL 500 mM hydrogen, peroxide according to the method of Aebi [19] with slight modifications. The assay was monitored for 4 min (20 s interval) at 240 nm using a spectra Max plate reader (Molecular Devices, CA, USA) and the results were expressed as μmol of H_2_O_2_ consumed/min/mg protein.

Glutathione-S-transferase activity was estimated by modifying the method of Habig and Jakoby [20]. The system had 135 μL 0.1 M potassium phosphate buffer (pH 6.5), 50 μL of tissue sample (0.2 mg/ml protein), 100 μL 3 mM glutathione, and 15 μL 20 mM CDNB. The spectra Max plate reader was set at 340 nm, 18 min (30 s interval) and results were expressed as μmol/min/mg protein.

### Statistical analyses

Data were expressed as mean ± standard error of mean. One-way analysis of variance (ANOVA) and Tukey’s post hoc test were utilized for data analyses and significance was considered at p < 0.05.

### Results

#### Acetylcholinesterase (AChE), redox and antioxidant activities of cockroaches exposed to 4% MSG and 1% MSG for 30 days

**Fig. 1:**
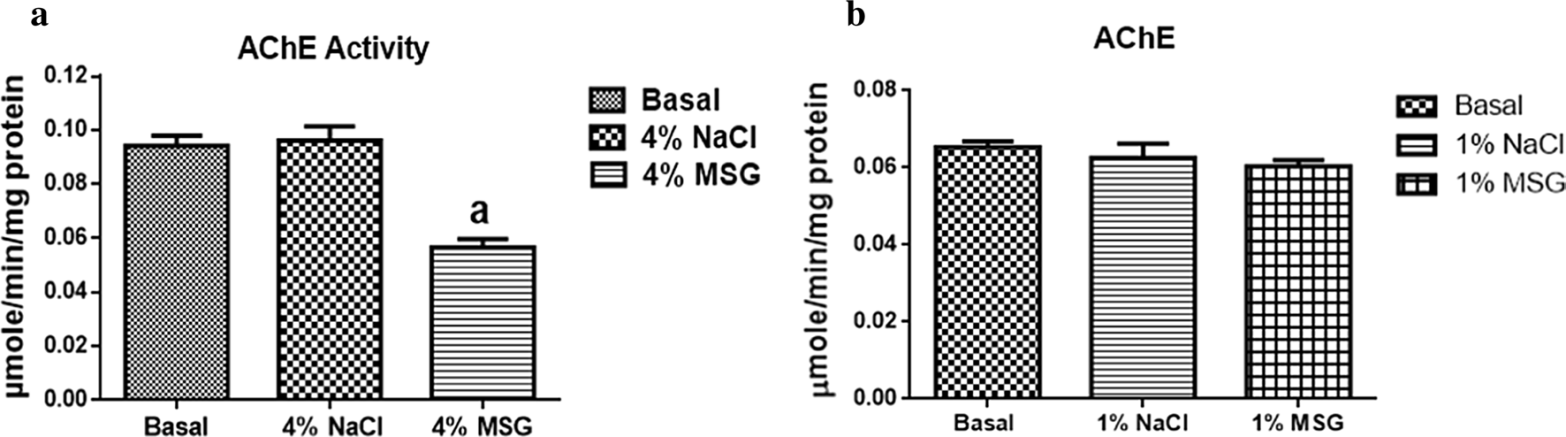
Acetylcholinesterase (AChE) activities of nymphs exposed to 4% MSG (**a**) and 1% MSG (**b**). The data are expressed as mean ± standard error mean (SEM). (a) differs significantly from the basal (p < 0.05)

**Fig. 2:**
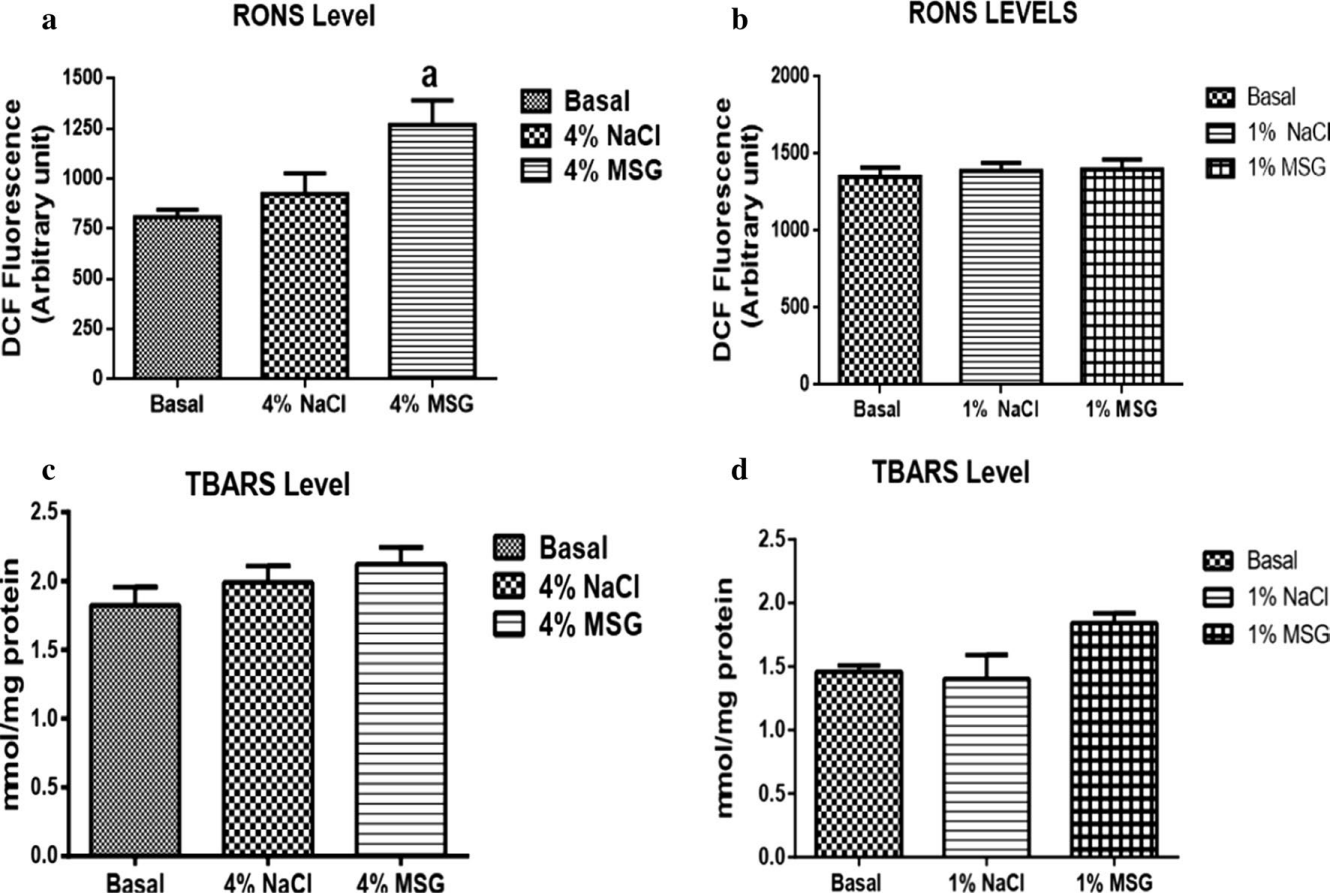
Oxidative stress markers in head homogenate of nymphs exposed to 4% MSG and 1% MSG. **a**, **b** RONs levels. **c**, **d** TBARs levels. The data are expressed as mean ± standard error mean (SEM). (a) differs significantly from the basal (p < 0.05)

**Fig. 3:**
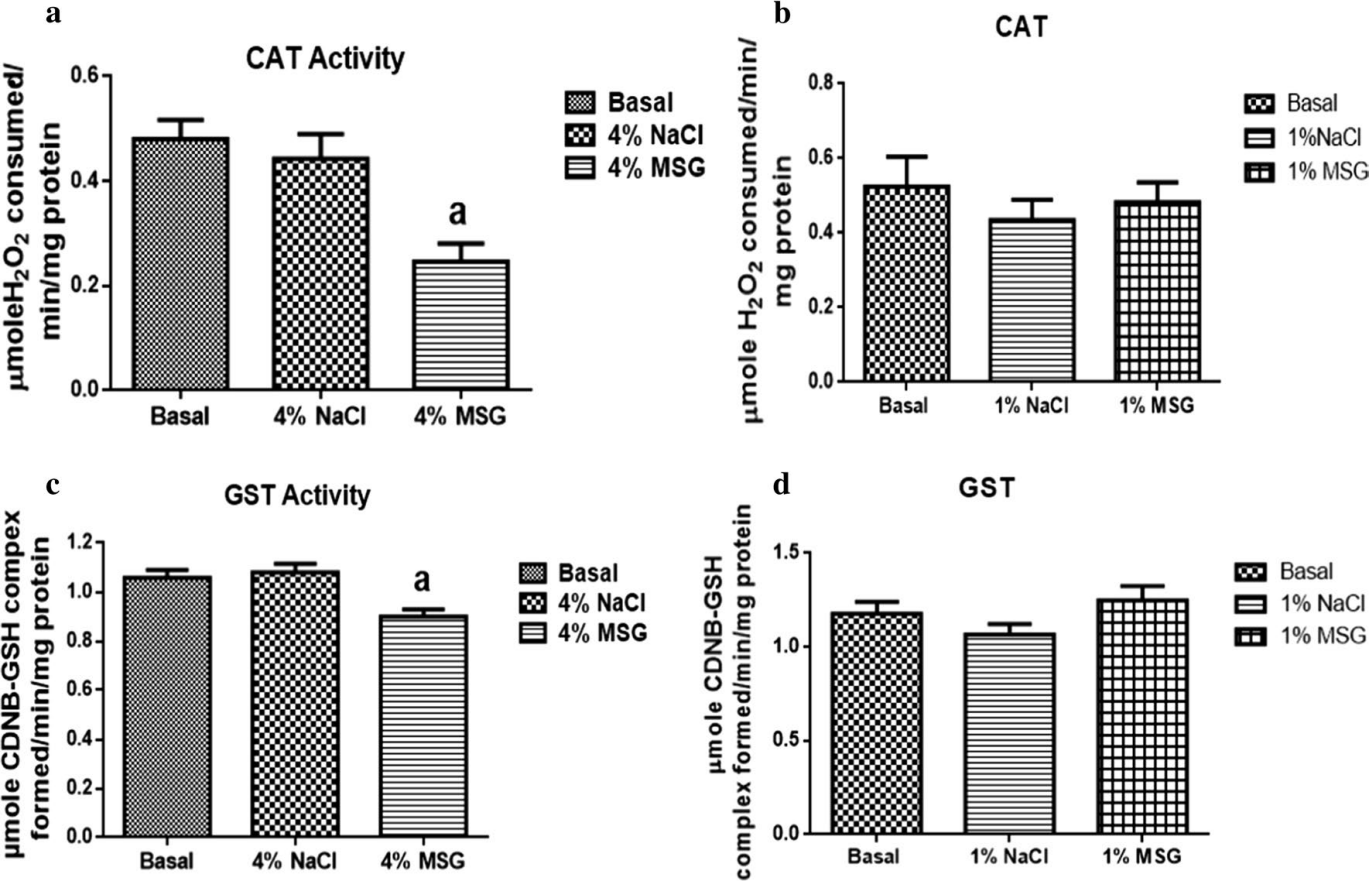
Antioxidant levels in head homogenate of nymphs exposed to 4% MSG and 1% MSG. **a**, **b** Catalase activities. **c**, **d** Glutathione-S-transferase (GST) activities. The data are expressed as mean ± standard error mean (SEM). (a) differs significantly from the basal (p < 0.05)

There was no significant difference in AchE (Fig. 1b), redox (Fig. 2b, d) and antioxidant activities (Fig. 3b, d) in cockroaches exposed to 1% MSG. Cockroaches exposed to diets containing 4% MSG showed a significant decrease in AChE activity by 40% (Fig. 1a), significant increase in RONS activity by 42% (Fig. 2a), no significant difference in TBARS levels (Fig. 2c), significant decrease in CAT activity by 49% (Fig. 3a), and significant decrease in GST activity by 15% (Fig. 3c) when compared to the basal group (control).

### Discussion

There has been widespread use of alternative models in neuroscience to assess the safety and toxicity of chemical substances [21, 22]. *N. cinerea,* a valid alternative model organism for basic toxicological studies has been reported to offer new insights for translational neuroscience research [12]. MSG has been established as a neurotoxicant in rodents and has recently been demonstrated to induce an adaptive response to oxidative stress in *Drosophila melanogaster* [8, 23, 24]. The present study reports some biochemical endpoints in the *N. cinerea* model following exposure to diets containing 4% MSG and 4% NaCl as well as, 1% MSG and 1%NaCl for 30 days.

Acetylcholinesterase (AChE) hydrolyses acetylcholine at synapses into thiocholine and acetic acid, thus playing a role in cholinergic neurotransmission as a biomarker for evaluating the functioning of the nervous system and the diagnosis of neurodegenerative diseases. Our study showed 4% MSG-treated cockroaches presented decreased AChE activity. Neurotransmission in the insect brain has both cholinergic and glutamatergic components [25, 26] and findings by Ortuño-Sahagún et al. [27] depict cholinergic interneurons in the cerebral cortex as major targets for glutamate—the main excitatory neurotransmitter in insects. It is therefore plausible that the administration of a glutamate source would enhance acetylcholine availability probably by suppressing AChE activity.

Chromosomal damage, as well as increased generation of reactive oxygen and nitrogen species are widely reported in insects exposed to MSG [8, 28, 29]. We equally found significant increase in RONS generation in 4% MSG treated cockroaches, though TBARS levels were unaffected. The brain is known to be susceptible to free radical damage because of its high concentration of polyunsaturated fatty acids, relatively low antioxidant capacity, high rate of oxygen use, and high concentration of transition metals in some of its’ regions [30]. Reports differ on the effect of MSG on *Drosophila melanogaster* antioxidant response [8, 9] we herein show reduced CAT and GST activities in *N. Cinerea* exposed to 4% MSG, suggesting that MSG might overwhelm the antioxidant and detoxification capabilities of insects. It is clear that at high concentration, MSG may disrupt antioxidant defence systems, nevertheless, nymphs exposed to 1% MSG showed no significant alterations in all parameters evaluated. We hypothesise that MSG is not toxic at low concentrations in the *N. cinerea* model, hence the safety of small concentrations of MSG for human consumption, even though clinical studies on MSG toxicity are scanty in the literature.

## Limitation

More information to establish the outcome of this study would be acquired if more antioxidant enzyme activities and glutamate levels were assessed in the heads of nymphs of exposed cockroaches. Moreover, this study shows basic information on the likely toxic effect of high concentration of MSG based on the activities of the antioxidant enzymes and primary cholinesterase (AChE) evaluated.

## Data Availability

The data used and analyzed during the present study are available from BAA on reasonable request.

## Abbreviations

MSG: Monosodium glutamate
CAT: Catalase
GST: Glutathione-S-transferase
AChE: Acetylcholinesterase
NaCl: Sodium chloride
M: Molar
mM: Millimolar
μL: Microliter
g: Relative centrifugal force

## References

[1] Ikeda K (2002). New seasonings. Chem Senses.

[2] Geha RS, Beiser A, Ren C, Patterson R, Greenberger PA, Grammer LC, Ditto AM, Harris KE, Shaughnessy MA, Yarnold PR, Corren J (2000). A review of alleged reaction to monosodium glutamate and outcome of a multicenter double-blind placebo-controlled study. J Nutr.

[3] He K, Du S, Xun P, Sharma S, Wang H, Zhai F, Popkin B (2011). Consumption of monosodium glutamate in relation to incidence of overweight in Chinese adults. China health and nutrition survey (CHNS). Am J Clin Nutr.

[4] Shivasharan BD, Nagakannan P, Thippeswamy BS, Veerapur VP (2013). Protective effect of Calendula officinalis L. flowers against monosodium glutamate induced oxidative stress and excitotoxic brain damage in rats. Ind J Clin Biochem.

[5] Farombi EO, Onyema OO (2006). Monosodium glutamate: induce oxidative damage and excitotoxicity in the rat. Modulatory role of vitamin C, vitamin E and quercetin. Hum Exp Toxicol.

[6] Onaolapo OJ, Onaolapo AY, Akanmu MA, Gbola O (2016). Evidence of alterations in brain structure and antioxidant status following ‘low-dose’ monosodium glutamate ingestion. Pathophysiology.

[7] Paul MV, Abhilash M, Varghese MV, Alex M, Nair RH (2012). Protective effects of alpha-tocopherol against oxidative stress related to nephrotoxicity by monosodium glutamate in rats. Toxicol Mech Methods.

[8] Abolaji AO, Olaiya CO, Oluwadahunsi OJ, Farombi EO (2017). Dietary consumption of monosodium l-glutamate induces adaptive response and reduction in the lifespan of *Drosophila Melanogaster*. Cell Biochem Funct.

[9] Kasozi KI, Namubiru S, Kiconco O, Kinyi HW, Ssempijja F, Ezeonwumelu JOC, Ninsiima HI, Okpanachi AO (2018). Low concentrations of monosodium glutamate (MSG) are safe in male *Drosophila melanogaster*. BMC Res Notes.

[10] Afolabi BA, Isaac AA, Diogo OS, João BTR (2018). Dietary co-exposure to methylmercury and monosodium glutamate disrupts cellular and behavioral responses in the lobster cockroach, *Nauphoeta Cinerea* Model. Environ Toxicol Pharmacol.

[11] Rodrigues NR, Nunes ME, Silva DG, Zemolin AP, Meinerz DF, Cruz LC, Pereira AB, Rocha JB, Posser T, Franco JL (2013). Is the lobster cockroach *Nauphoeta cinerea* a valuable model for evaluating mercury induced oxidative stress?. Chemosphere.

[12] Zemolin AP, Cruz LC, Paula MT, Pereira BK, Albuquerque MP, Victoria FC, Pereira AB, Posser T, Franco JL (2014). Toxicity induced by *Prasiola crispa* to Fruit Fly *Drosophila melanogaster* and Cockroach *Nauphoeta cinerea*: Evidence for bioinsecticide action. J Toxicol Environ Health A.

[13] Afolabi BA, Olagoke OC, Souza DO, Aschner M, Rocha JB, Segatto AL (2020). Modified expression of antioxidant genes in lobster cockroach, Nauphoeta cinerea exposed to methylmercury and monosodium glutamate. Chemico Biological Interact.

[14] Stankiewicz M, Da Browski M, De Lima ME (2012). Nervous system of *Periplaneta americana* cockroach as a model in toxinological studies: a short historical and actual view. J Toxicol.

[15] Harris WE, Moore PJ (2005). Female mate preference and sexual conflict: females prefer males that have had fewer consorts. Am Nat.

[16] Ellman GL (1959). Tissue sulfhydryl groups. Arch Biochem Biophys.

[17] Piccoli BC, Alvim JC, da Silva FD, Nogara PA, Olagoke OC, Aschner M, Oliveira CS, Rocha JBT (2020). High level of methylmercury exposure causes persisted toxicity in *Nauphoeta cinerea*. Environ Sci Pollut Res.

[18] Puntel RL, Roos DH, Grotto D, Garcia SC, Nogueira CW, Rocha JBT (2020). Antioxidant properties of Krebs cycle intermediates against malonate pro-oxidant activity in vitro: a comparative study using the colorimetric method and HPLC analysis to determine malondialdehyde in rat brain homogenates. Life Sci..

[19] Aebi H (1984). Catalase in vitro. Methods Enzymol.

[20] Habig WH, Jakoby WB (1981). Assays for differentiation of glutathione S-transferases. Methods Enzymol.

[21] Schechtman LM (2002). Implementation of the 3Rs (refinement, reduction, and replacement) validation and regulatory acceptance considerations for alternative toxicological test methods. ILAR J.

[22] Peterson RT, Nass R, Boyd WA (2008). Use of non-mammalian alternative models for neurotoxicological study. Neurotoxicology.

[23] Olney JW (1969). Brain lesions, obesity, and other disturbances in mice treated with monosodium glutamate. Science.

[24] Kubo T, Kohira R, Okano T, Ishikawa K (1993). Neonatal glutamate can destroy the hippocampal CA1 structure and impair discrimination learning in rats. Brain Res.

[25] Kratsios P, Stolfi A, Levine M, Hobert O (2012). Coordinated regulation of cholinergic motor neuron traits through a conserved terminal selector gene. Nat Neurosci.

[26] Stein W, Smarandache CR, Nickmann M, Hedrich UBS (2006). Functional consequences of activity-dependent synaptic enhancement at a crustacean neuromuscular junction. J Exp Biol.

[27] Ortuño-Sahagún D, Beas-Zárate C, Adame-Gonzalez G, Feria-Velasco A (1997). Effect of l-glutamate on cholinergic neurotransmission in various brain regions and during the development of rats, when administered perinatally. Neurochem Int.

[28] Ataseven N, Yüzbaşioğlu D, Keskin AÇ, Ünal F (2016). Genotoxicity of monosodium glutamate. Food Chem Toxicol.

[29] El-Keredy A (2014). Genetic and behavioural influences of quinine and monosodium glutamate on *Drosophila melanogaster*. Egypt J Genet Cytolo.

[30] Evans PH (1993). Free radicals in brain metabolism and pathology. Br Med Bull.

